# Introduction of Extracorporeal Membrane Oxygenation on Ninth Day of Ventilator Management for Severe Acute Respiratory Distress Syndrome due to COVID-19: A Case Report

**DOI:** 10.7759/cureus.24869

**Published:** 2022-05-10

**Authors:** Akiko Takeda, Michiaki Higashitani, Yuko Kuroda, Kentaro Ohgi, Kunio Yanagita

**Affiliations:** 1 Intensive Care, Tokyo Medical University Ibaraki Medical Center, Ibaraki, JPN; 2 Cardiology, Tokyo Medical University Ibaraki medical center, Ibaraki, JPN

**Keywords:** ventilator management, timing of ecmo introduction, extracorporeal membrane oxygenation, acute respiratory distress syndrome, covid-19

## Abstract

The reported case is of a 68-year-old man who was admitted to the ICU at our tertiary care medical center with severe COVID-19. He was admitted to the ICU due to a worsening respiratory condition during his hospitalization at the same medical center, which included the development of severe acute respiratory distress syndrome (ARDS). Ventilator management was started with alveolar protection in mind. On the ninth day of ventilator management, we judged that it was necessary to introduce extracorporeal membrane oxygenation (ECMO). Although the ninth day of ventilator management is considered relatively late for starting ECMO, there are no absolute contraindications for ECMO at this stage, and improvements in oxygenation can be expected. After introducing ECMO, the patient’s oxygenation capacity improved, and ECMO was successfully withdrawn within 16 days. The patient required long-term rehabilitation but was discharged from the hospital to his home without lingering disease complications on the 150th day of illness and subsequently resumed his former work, daily activities, and quality of life. We conclude that, in regard to the introduction of ECMO for ARDS, it is necessary to reach a comprehensive judgment without being bound by any one index (such as the ventilation management period prior to ECMO introduction).

## Introduction

The coronavirus disease 2019 (COVID-19) is reported to have a severe presentation in 14% of affected cases and to require intensive care in 5% of affected cases; approximately half of individuals requiring intensive care for COVID-19 die due to this illness [1]. Moreover, approximately 75% of critically ill patients requiring treatment in the ICU require mechanical ventilation and present with acute respiratory distress syndrome (ARDS) [2]. Acute respiratory distress syndrome is an extremely severe condition requiring intensive treatment in severe cases of COVID-19.

Various primary diseases, such as sepsis and trauma, underly the development of ARDS [3]. The effectiveness of extracorporeal membrane oxygenation (ECMO), an intensive respiratory treatment, for severe ARDS arising from various primary diseases has been investigated previously [4,5]. A prior retrospective study reported that ECMO treatment for severe ARDS associated with COVID-19 had the same positive effects as ECMO treatment for ARDS associated with a primary underlying disease other than COVID-19 [6]. However, studies have likewise reported an extremely high mortality rate in cases in which ventilator management before the introduction of ECMO lasts for six to seven days or more [7,8].

The Extracorporeal Life Support Organization (ELSO), an international research organization focusing on ECMO, published guidelines for ECMO treatment in cases of severe ARDS associated with COVID-19 in 2020. These guidelines noted that ECMO is contraindicated in cases where ventilator management exceeds 10 days [9]. However, we conducted an extensive literature search on this topic to inform the current case report and did not find any case reports or clinical studies comprehensively examining the indications for the introduction of ECMO as well as for the timing of its introduction in patients with severe ARDS occurring due to COVID-19.

Herein, we report a case of severe ARDS caused by COVID-19 in which ECMO introduction on the ninth day of ventilator management proved successful and lifesaving.

## Case presentation

The presenting case was a Japanese 68-year-old male (170 cm height, 84 kg weight, 29.0 kg/m2 body mass index [BMI]) in April 2020 without variant SARS-Cov2. His initial chief complaint was a sore throat and fever. He had a personal medical history of diabetes, hypertension, and hyperlipidemia. He had quit smoking at the age of 50 after smoking 30 cigarettes/day for 30 years, and drank socially. He had not been vaccinated against SARS-Cov2. 

His current medical history at the time of hospital admission included strong activities of daily living (ADL) scores, and he was gainfully employed at the time of hospital admission. He complained of a sore throat that had appeared three days before his hospital admission and a fever of 38.4°C that had appeared two days before hospital admission. He had been in contact with a COVID-19 patient at work, and a chest CT scan showed evidence of pneumonia. Therefore, a PCR sample (reverse transcription-polymerase chain reaction; SARS-CoV-2 RT-PCR) was collected and he was hospitalized urgently.

His symptoms on initial hospital admission were as follows: full response on the Glasgow coma scale, a blood pressure of 148/103 mmHg, a pulse of 90 beats/minute, oxygen saturation of 93% on room air, a body temperature of 38.6°C, and a respiratory rate 20 breaths/minute. He showed no evidence of respiratory distress and no lung noise.

His blood sampling data were as follows: leukocytes at 7,500/μL, platelets at 13.2 million/μL, aspartate aminotransferase (AST) of 118 U/L, alanine transaminase (ALT) of 115 U/L, lactate dehydrogenase (LDH) at 458 U/L, creatine kinase (CK) at 2,938 U/L, blood urea nitrogen (BUN) at 15.1 mg/dL, creatinine (Cr) of 1.06 mg/dL, C-reactive protein (CRP) of 4.57 mg/dL, and a fibrin degradation product (FDP) level of 2.6 μg/mL.

His clinical course following hospital admission included oxygen administration for respiratory failure (at a rate of 3 L/min via an oxygen mask) that was started at the time of admission. On the second day of hospitalization, his PCR test came back positive and the patient was formally diagnosed with COVID-19. His respiratory condition subsequently worsened, and the oxygen dose was increased to cope with his worsening condition. Ultimately, he underwent tracheal intubation in the ward and entered the ICU.

His sepsis-related organ failure assessment (SOFA) score at the time of admission to the ICU was 7 points, and his acute physiology and chronic health evaluation (APACHE) II score was 31 points, indicating a poor prognosis (APACHE II score estimated mortality rate, 73.2%). Infiltration shadows on both lung fields were observed on the chest X-ray, and the patient’s ratio of arterial oxygen partial pressure to fractional inspired oxygen (PaO2/FiO2 or, P/F ratio) was 105.6 at a plateau pressure of 30 cmH2O. Consequently, ARDS was diagnosed according to established medical guidelines [3].

Three-day pulse therapy with 1000 mg of methylprednisolone was performed on the fourth day of illness (i.e., hereinafter referred to in terms of days of hospitalization), and the P/F ratio on the seventh day of illness improved to 215.7. On the seventh day of illness, 80 mg/day of prednisolone was administered as maintenance therapy; this dose was gradually reduced thereafter.

Ventilator management aims to keep plateau pressure below 30 cmH2O with high positive end-expiratory pressure (PEEP), along with a PaO2 index of 60 mmHg or higher and a FiO2 of 0.6 or lower to prevent alveolar injury due to ARDS [1,3,5]. Tube feeding was started as the patient’s nutritional management on the fifth day of illness. There was an increase in FDP (7.4 μg/mL) on the seventh day of illness, and we, therefore, judged that there was a high risk of deep vein thrombosis due to long-term bed rest, and central venous indwelling, and severe infection. Hence, the patient was prescribed unfractionated heparin sodium (12,000 units). His CRP levels decreased initially but started to increase again on the 11th day of illness, and polymyxin B-immobilized fiber column direct hemoperfusion (PMX-DHP) for systemic inflammatory response syndrome (SIRS) due to hypercytokinemia associated with ARDS and COVID-19 was performed. Following this, his treatment shifted to continuous hemodiafiltration (CHDF). However, his respiratory status worsened again and his PEEP was increased to 14 cmH2O. Although he was under sedation and analgesia therapy, he had a strong cough reflex. On the ninth day of illness, cough reflex-induced mediastinal emphysema appeared on a chest X-ray and showed a tendency to expand despite lowering the plateau and peak pressure setting of the ventilator as much as possible. Judging that it would be difficult to treat his condition with ventilator management only, ECMO was introduced on the 12th day of illness (the ninth day after the start of ventilator management) (see Table 1). The 21Fr Drainage cannula was inserted into the right femoral vein and the 16.5Fr return cannula into the right internal jugular vein. When ECMO was started, the settings were 3400 rpm, 4.2 l/min blood flow, and FIO2 of 0.8. 

**Table 1:** Progress chart from the time of entering the ICU to the introduction of extracorporeal membrane oxygenation (ECMO) mPSL: methylprednisolone, PaCO2: Pressure of arterial carbon dioxide, PSL: Prednisolone, PEEP: Positive end-expiratory pressure, pH: Potential of hydrogen, P/F ratio: Ratio of arterial oxygen partial pressure (PaO2 in mmHg) to fractional inspired oxygen (FiO2)

	Day 4	Day 5	Day 6	Day 7	Day 8	Day 9	Day 10	Day 11	Day 12
P/F ratio	105.6	135	146.1	215	185.4	129.8	151	133	137
PaCO2	60.7	41.1	43.2	45.9	49.9	46	45.5	41.9	41.5
pH	7.26	7.373	7.391	7.412	7.421	7.425	7.413	7.438	7.412
PEEP	10	10	10	12	12	12	14	12	12
						Mediastinal emphysema			
	Ventilator management								
	mPSL 1000mg/day			PSL 80mg/day					
									ECMO Introduction

An improvement in oxygenation ability was observed starting on the 23rd day of illness (i.e., the 12th day of ECMO), and ECMO was successfully withdrawn on the 27th day of illness. A tracheostomy was performed on the 37th day of illness, and a chest CT taken on the same day showed that the frosted glass shadow of the lung field was enhanced and enlarged compared to that seen at the time of admission. After the tracheotomy, sedation was discontinued and the patient gradually awakened and started to take food and medication orally. Continuous hemodiafiltration continued until the 44th day of illness due to the appearance of renal dysfunction following the introduction of ECMO.

**Figure 1:**
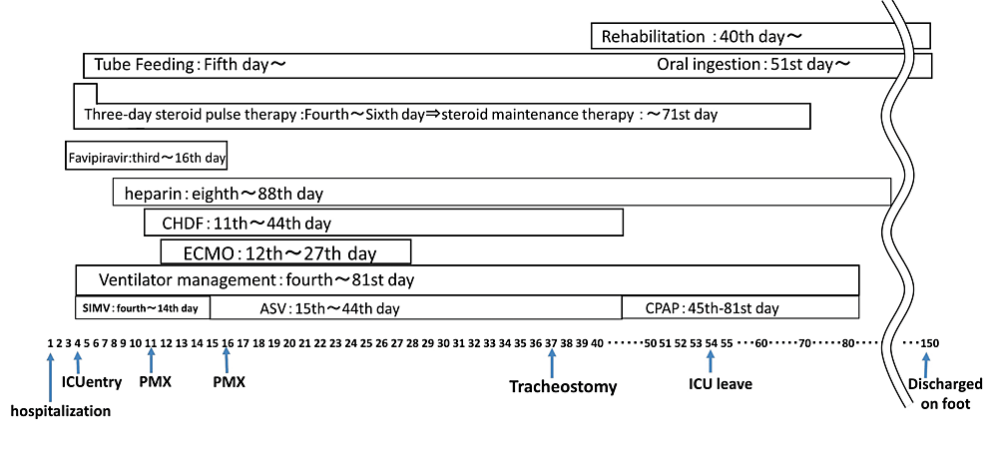
Hospitalization progress chart depicting the patient’s clinical course. ASV: Adaptive support ventilation, CPAP: Continuous positive airway pressure, SIMV: Synchronized intermittent mandatory ventilation, PMX: Polymyxin B hemoperfusion

**Figure 2:**
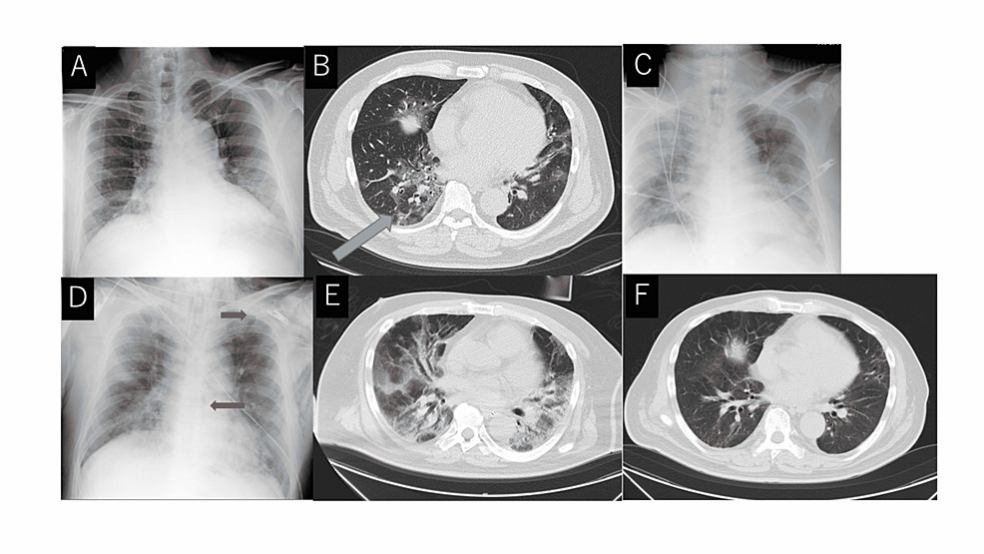
Progressive chest X-ray and chest CT findings A: Chest X-ray on admission, B: Chest CT on admission; a frosted glass shadow was observed in the center of the lower leaves on both sides of the image,  C: Chest X-ray on ICU admission; an infiltration shadow was seen in the bilateral lung fields, D: Chest X-ray after starting extracorporeal membrane oxygenation (ECMO). Mediastinal emphysema and subcutaneous emphysema were observed, E: Chest CT at 34 days of hospitalization (11 days after ECMO withdrawal). Frosted glass shadows were observed throughout the lung field, F: Chest CT at 149 days (prior to hospital discharge); frosted glass shadows remained on both lower lobes but were significantly improved.

Rehabilitation was started on the 40th day of illness. However, the patient’s respiratory muscle fatigue was strong. On the 54th day of illness, he was discharged from the ICU with the ventilator attached; he withdrew from the ventilator on the 81st day of illness and the tracheostomy was closed on the 108th day of illness (see Figure 1). On the 150th day of illness, he was discharged from the hospital without the need for home oxygen therapy. His symptom of numbness in the fingers remained. However, he ultimately successfully returned to work and society without any problems in his daily life. Figure 2 shows the progress of chest X-ray and chest CT

## Discussion

The most recent ELSO guidelines state that ECMO should be considered given a predicted mortality rate of 50% or higher and should certainly be introduced at a predicted mortality rate of 80% or higher (despite appropriate and adequate treatment for the reversible disease) [9]. In the reported case, the patient’s age (i.e., 65 years or older) applied to the relative ECMO maladaptation criteria specified in the guidelines. However, absolute ECMO maladaptation criteria, such as frailty progression, uncontrolled diabetes, bleeding complications, dementia, and ventilator management for 11 days or more did not apply to the current case. In addition, if the deviation of the ventilator setting from the viewpoint of alveolar protection in ARDS (i.e., plateau pressure exceeding 30 cmH2O or FiO2 exceeding 60%) continues for seven days or more, ECMO introduction is considered to be maladaptive by current guidelines [5]. Following one day during which both plateau pressure and FiO2 deviated from the standard, we determined that the application of ventilator management with due attention to alveolar protection had been achieved and that a reversal to the attained improvement in lung function could be expected. It has been reported that lung-protective ventilatory management should be performed thoroughly during ECMO in severe COVID-19 pneumonia with pneumomediastinum/subcutaneous emphysema [10]. We have to keep in mind lung-protective ventilatory management not only during ECMO but also before ECMO introduction in severe COVID-19 pneumonia.

Therefore, we decided to introduce ECMO to our 68-year-old patient although it was relatively late in the ventilator management course (i.e., ECMO was introduced on the ninth day of ventilator management). We note that subcutaneous emphysema, which is one of the criteria for introducing ECMO according to the 2020 ELSO guidelines, appeared on the sixth day of ventilator management. Accordingly, ECMO should have been introduced on the next day at the latest, and ideally should have been introduced on the same day. The first reason for the delay was the novelty of this case at our hospital and the fact that there was a lack of awareness about the introduction of ECMO at our tertiary care center, even though the patient’s prognosis was predicted to be poor when entering the ICU. The other reason for the delay was that the CT findings on admission had a low severity score, and the blood gas findings were maintained to some extent. [11].

The results of the extensive multicenter ECMO to Rescue Lung Injury in Severe ARDS (EOLIA) trial, a randomized controlled trial that was designed to verify the efficacy of ECMO in patients with severe ARDS, have been reported previously [12]. In this study, patients with severe ARDS who met certain respiratory failure conditions (i.e., for whom ECMO could be considered) were randomly divided into groups receiving ECMO induction and ECMO avoidance treatment conditions; this allocation was randomized to the extent possible. Patients’ mortality rates were compared after 60 days. More specifically, ECMO was introduced 6.5 days after randomization on average in 28% of the patients in the ECMO avoidance group. In other words, rather than directly verifying the effectiveness of ECMO for severe ARDS, the strategy of this trial was to introduce ECMO at an early stage of ventilator management (i.e., at approximately 1.5 days). If necessary, ECMO also was introduced to the avoidance group (eight days later on average). It can thus be said that this trial indirectly led to a comparative test of ECMO treatment as compared with ECMO avoidance. However, due to the early discontinuation of the study and the small number of enrolled patients, the effectiveness of the early ECMO introduction strategy could not be demonstrated conclusively. However, the confidence interval ranged from 0.55 to 1.04 (i.e., precise and near statistical significance), and the prognosis tended to be good overall in the early strategy group.

In the current case, the strategy employed at our hospital ICU was to introduce ECMO if necessary. Considering the results of the EOLIA study, this strategy is generally considered appropriate. However, in a Western multicenter registry wherein ECMO was introduced for ARDS associated with COVID-19, the survival prognosis deteriorated when the ventilator management period before the introduction of ECMO was prolonged (and ECMO was ultimately required) [13]. Thus, evidence to date strongly suggests that ECMO should be introduced early.

We also consider the disadvantages of introducing ECMO herein. It has been reported that approximately 20% of direct complications in the treatment of ARDS are caused by ECMO; moreover, approximately 20% of indirect complications include extremely serious events such as bleeding events and secondary infections [5]. Since any complication can be fatal, physicians should refrain from introducing unnecessary ECMO treatment. In ECMO treatment for ARDS, the patient's prognoses are improved in facilities treating 30 or more cases per year as compared with facilities treating fewer than 30 cases, and there is evidence that both clinical experience and information sharing about ECMO treatment are critically important in ensuring optimal patient outcomes [12].

Hence, based on the findings of our case report and according to the overall literature to date, we consider complications and adjuvant therapies to reduce the number of cases requiring the introduction of ECMO herein. For example, it has been reported that ventilator management in the prone position halves the 90-day mortality rate for severe ARDS [14]. In this case, the ventilator could not be managed in the prone position because the medical teams at our tertiary care center were not accustomed to infection control in the early stages of COVID-19 infection. However, the rate of prone position therapy for severe ARDS due to early COVID-19 in the United States has been reported to be approximately 28% according to the 2020 findings [2]. At our hospital, prone position therapy was performed to the extent possible after encountering our third case of severe COVID-19.

Moreover, another consideration is that systemic administration of steroids is expected to suppress lung injury caused by cytokines, and the effectiveness of dexamethasone in patients with COVID-19 who have higher oxygen demand has previously been reported [15]. In this case, the patient’s respiratory condition deteriorated rapidly, and pulse therapy was hence selected at the start of treatment administration. However, pulse therapy is not typically recommended for patients with COVID-19, even in severe cases [16]. Hence, comprehensive medical recommendations should be elucidated more thoroughly in future research.

Finally, with regard to severe ARDS occurring due to COVID-19 infection, we summarize our current thinking on the introduction of ECMO for patients based on our clinical experience as well as the findings of the literature to date herein. We conclude that physicians should strive to manage ventilators so as to protect the alveoli and to try to avoid the introduction of ECMO by applying various adjuvant therapies in combination. However, it is likewise important to establish a system that can assist in promptly introducing ECMO when a patient’s respiratory condition deteriorates. Moreover, when introducing ECMO, it is desirable to judge the ECMO introduction time appropriately and comprehensively without being bound by any one index (such as the ventilator management period).

## Conclusions

Herein we report a case of severe ARDS caused by COVID-19. Extracorporeal membrane oxygenation (ECMO) was introduced relatively late in the patient’s clinical course i.e., on the ninth day of ventilator management. This timing was not in direct contraindication to any of the current guidelines, though some current guidelines do discourage late administration of ECMO therapy and there are some specific guidelines in place in which ECMO administration is contraindicated when ventilator management exceeds 10 days. However, in our case, implementing the strategy of late ECMO administration proved to be both curative and lifesaving. Thus, we recommend that this topic be explored more comprehensively in rigorous clinical research and additional case reports and that medical guidelines be carefully considered about our findings as well as those of other conflicting case reports and investigations. We stress that the patient returned to his previous quality of life following this treatment course. Our work hence provides important evidence informing research directions, medical guidelines, and effective clinical decision-making.

## References

[1] Berlin DA, Gulick RM, Martinez FJ (2020). Severe Covid-19. N Engl J Med.

[2] Bhatraju PK, Ghassemieh BJ, Nichols M (2020). Covid-19 in critically ill patients in the Seattle region - case series. N Engl J Med.

[3] Thompson BT, Chambers RC, Liu KD (2017). Acute respiratory distress syndrome. N Engl J Med.

[4] Peek GJ, Mugford M, Tiruvoipati R (2009). Efficacy and economic assessment of conventional ventilatory support versus extracorporeal membrane oxygenation for severe adult respiratory failure (Cesar): a multicentre randomised controlled trial. Lancet.

[5] Brodie D, Bacchetta M (2011). Extracorporeal membrane oxygenation for ARDS in adults. N Engl J Med.

[6] Schmidt M, Hajage D, Lebreton G (2020). Extracorporeal membrane oxygenation for severe acute respiratory distress syndrome associated with COVID-19: a retrospective cohort study. Lancet Respir Med.

[7] Giraud R, Legouis D, Assouline B (2021). Timing of VV-ECMO therapy implementation influences prognosis of COVID-19 patients. Physiol Rep.

[8] Patroniti N, Zangrillo A, Pappalardo F (2011). The Italian ECMO network experience during the 2009 influenza A(H1N1) pandemic: preparation for severe respiratory emergency outbreaks. Intensive Care Med.

[9] Shekar K, Badulak J, Peek G (2020). Extracorporeal Life Support Organization coronavirus disease 2019 interim guidelines: a consensus document from an international group of interdisciplinary extracorporeal membrane oxygenation providers. ASAIO J.

[10] Kohara J, Kai S, Hashimoto K (2022). Successful lung-protective ventilatory management during the VV-ECMO in a severe COVID-19 pneumonia patient with extensive pneumomediastinum and subcutaneous emphysema: a case report. JA Clin Rep.

[11] Francone M, Iafrate F, Masci GM (2020). Chest CT score in COVID-19 patients: correlation with disease severity and short-term prognosis. Eur Radiol.

[12] Combes A, Hajage D, Capellier G (2018). Extracorporeal membrane oxygenation for severe acute respiratory distress syndrome. N Engl J Med.

[13] Lebreton G, Schmidt M, Ponnaiah M (2021). Extracorporeal membrane oxygenation network organisation and clinical outcomes during the COVID-19 pandemic in Greater Paris, France: a multicentre cohort study. Lancet Respir Med.

[14] Guérin C, Reignier J, Richard JC (2013). Prone positioning in severe acute respiratory distress syndrome. N Engl J Med.

[15] Horby P, Lim WS, Emberson JR (2021). Dexamethasone in hospitalized patients with Covid-19. N Engl J Med.

[16] Bhaskar S, Sinha A, Banach M (2020). Cytokine storm in COVID-19-immunopathological mechanisms, clinical considerations, and therapeutic approaches: the REPROGRAM consortium position paper. Front Immunol.

